# GENERATIVITY AND OLDER ADULTS’ COVID-19 ADJUSTMENT AND MENTAL HEALTH

**DOI:** 10.1093/geroni/igac059.1898

**Published:** 2022-12-20

**Authors:** Christine So, Katherine Fiori, Amy Rauer, Christina Marini

**Affiliations:** Adelphi University, Garden City, New York, United States; Adelphi University, Garden City, New York, United States; University of Tennessee Knoxville, Knoxville, Tennessee, United States; Adelphi University, Garden City, New York, United States

## Abstract

Generativity, the capacity to be productive, caring, and concerned with the well-being of the next generation, has been linked to positive mental health outcomes and posttraumatic growth (Bellizzi, 2004). Generativity may be particularly important nowadays as older adults adjust to the pandemic and its aftermath. For example, after months of social distancing, the availability of vaccines has enabled many older adults to begin resuming social activities. Considering the meaning-making function of generativity, generativity may be associated with more positive outcomes, including better mental health and views on quality of life and family relationships during this post-pandemic adjustment period. The current study used a community sample of 136 older adults (M age = 67.77, range 50-91; 69.3% females; 93% White) to explore whether generativity predicted older adults’ anxiety and depressive symptoms, and attitudes about how the pandemic affected their quality of life and family relationships. Using hierarchical linear regressions controlling for age and gender, we found that generativity was negatively linked to anxiety and depressive symptoms. Furthermore, those with greater generativity were more likely to report that their family relationships improved because of the pandemic. In contrast, generativity was not associated with positive growth in the personal domain or with perceptions that the pandemic had harmed either personal or family domains. Our findings are consistent with Erikson’s theory on the important role that generativity plays in shaping well-being and psychological health in older adults, and our findings suggest these effects may be especially pronounced during this post-pandemic adjustment period.

